# Lobaplatin arrests cell cycle progression in human hepatocellular carcinoma cells

**DOI:** 10.1186/1756-8722-3-43

**Published:** 2010-10-31

**Authors:** Qiong Wu, Shu-Kui Qin, Feng-Meng Teng, Chang-Jie Chen, Rui Wang

**Affiliations:** 1 Department of Medical Oncology, Affiliated Hospital of Bengbu Medical College, Bengbu, Anhui, China; 2 Department of Oncology, the 81 Hospital of the Chinese People's Liberation Army, Nanjing, China; 3 Department of Laboratory Medicine, Bengbu Medical College, Bengbu, Anhui, China

## Abstract

**Background:**

Hepatocellular carcinoma (HCC) still is a big burden for China. In recent years, the third-generation platinum compounds have been proposed as potential active agents for HCC. However, more experimental and clinical data are warranted to support the proposal. In the present study, the effect of lobaplatin was assessed in five HCC cell lines and the underlying molecular mechanisms in terms of cell cycle kinetics were explored.

**Methods:**

Cytotoxicity of lobaplatin to human HCC cell lines was examined using MTT cell proliferation assay. Cell cycle distribution was determined by flow cytometry. Expression of cell cycle-regulated genes was examined at both the mRNA (RT-PCR) and protein (Western blot) levels. The phosphorylation status of cyclin-dependent kinases (CDKs) and retinoblastoma (Rb) protein was also examined using Western blot analysis.

**Results:**

Lobaplatin inhibited proliferation of human HCC cells in a dose-dependent manner. For the most sensitive SMMC-7721 cells, lobaplatin arrested cell cycle progression in G_1 _and G_2_/M phases time-dependently which might be associated with the down-regulation of cyclin B, CDK1, CDC25C, phosphorylated CDK1 (pCDK1), pCDK4, Rb, E2F, and pRb, and the up-regulation of p53, p21, and p27.

**Conclusion:**

Cytotoxicity of lobaplatin in human HCC cells might be due to its ability to arrest cell cycle progression which would contribute to the potential use of lobaplatin for the management of HCC.

## Background

Hepatocellular carcinoma (HCC) is one of the most common cancers with poor prognosis. In China alone, more than 401,000 new patients were diagnosed with HCC and more than 371,000 patients died of this disease in 2008 [1]. The poor outcome of HCC is mainly due to it rarely presents with characteristic symptoms at early stage and over 80% of patients lose the chance of curative hepatectomy when the diagnosis of HCC was confirmed [2].

For the management of advanced HCC, systemic chemotherapy with classical cytotoxic agents offers a marginal survival benefit [3,4]. To improve the chemotherapeutic efficacy, a few of novel cytotoxic agents have been employed to treat patients with HCC. Oxaliplatin, a third-generation platinum compound, has exhibited promising activity against advanced HCC with tolerable toxicity in phase II clinical trials [5,6]. Recently, a randomized controlled phase III trial has been performed to evaluate the efficacy of FOLFOX4 (oxaliplatin plus 5-fluorouracil/leucovorin) in Asian patients with advanced HCC. The data from first interim analysis have shown a significant advantage of FOLFOX4 over doxorubicin in terms of overall response rate (ORR), disease control rate (DCR), and time to progression (TTP) [7].

As another third-generation platinum compound, lobaplatin (D-19466; 1, 2-diammino-methyl-cyclobutaneplatinum(II)-lactate) has shown encouraging anti-cancer activity in a variety of tumor types without evident hepatotoxicity [8-10] and has been approved in China for the treatment of chronic myelogenous leukemia (CML), metastatic breast cancer and small cell lung cancer [11]. It is noteworthy that some tumors resistant to cisplatin are still sensitive to lobaplatin [8]. Base on these considerations, we speculate lobaplatin might be useful for advanced HCC patients but more experimental and clinical data are warranted. In the present study, the effect of lobaplatin was assessed in five human HCC cell lines and the underlying molecular mechanisms in terms of cell cycle kinetics were explored.

## Materials and methods

### Cell culture

Lobaplatin and oxaliplatin were purchased from Hainan Chang'an International Pharmaceutical (Hainan, China) and Sigma (St. Louis, MO, USA), respectively. The human HCC cell lines, SMMC-7721, Bel-7402, HepG2, and Huh-7, were obtained from the Institute of Biochemistry and Cell Biology, Chinese Academy of Sciences (Shanghai, China). Hep 3B was kindly provided by Dr. X. Wang (Department of Oncology, Changzheng Hospital, Shanghai, China). All cell lines were maintained in Dulbecco's modified Eagle's medium (Gibco BRL, Carlsbad, CA, USA) supplemented with 10% fetal bovine serum (Gibco) at 37°C in a humidified atmosphere containing 5% CO_2_.

### Proliferation assay

Cytotoxicity of lobaplatin to human HCC cell lines was examined using cell proliferation assay. Cells were seeded in a 96-well microtiter plate at 5 × 10^3 ^cells/well, and cultured for 24 hours prior to exposure to lobaplatin or oxaliplatin of varying concentrations for 48 hours. Ten μl 3-(4, 5-dimethylthiazol-2-yl)-2, 5-diphenyltetrazolium bromide (MTT, 5 mg/ml) in phosphate buffered saline (PBS) were then added to each well. Four hours later the culture media was discarded and the dark blue crystals were dissolved in 100 μl dimethylsulfoxide (DMSO). The optical density (OD) was measured at 560 nm using a microplate reader (Thermo labsystems, Helsinki, Finland). Six wells were used for each concentration. The 50% inhibitory concentration (IC_50_) was calculated by nonlinear regression fit of the mean values of the data obtained in triplicate independent experiments.

### Flow cytometric (FCM) analysis

The effect of lobaplatin on human HCC cell cycle distribution was determined by FCM analysis. Cells were seeded in six-well plates at 5 × 10^5 ^cells/well and cultured for 24 hours prior to lobaplatin exposure for 0, 24, 36 and 48 hours. Control cells received only solvent for the indicated time durations above. Cells were collected by trypsinization, washed twice with ice cold PBS, fixed in 70% ethanol, and stained with propidium iodide (PI; 5 μg/ml PI in PBS containing 0.1% Triton X-100 and 0.2 mg/ml RNase A) overnight at 4°C in the dark until analyzed using a FACScan flow cytometer (BD Biosciences, San Jose, CA, USA). Cell fluorescence was measured in duplicate at each time point and all experiments were performed in triplicate.

### Reverse transcription polymerase chain reaction (RT-PCR) analysis

The mRNA expression of cell cycle-regulated genes was examined by RT-PCR. Total RNA was extracted using Trizol solution (Invitrogen, Carlsbad, CA, USA). Single-stranded cDNAs were synthesized with oligo (dT) primers in a reaction starting with 2 μg of total RNA using Superscript II reverse transcriptase (Fermentas Life Sciences, Hanover, MD, USA). PCR amplification was carried out in 25 μl total volume containing: 2 μl cDNA, 200 μM each dNTP, 0.25 units Taq polymerase, and 1 μM each primer (Sangon, Shanghai, China). Reaction conditions were optimized as follows: activation at 95°C for 5 min, followed by 30-35 cycles at 94°C for 45 s, 55-64°C for 45 s, and 72°C for 1 min. A series of calibration experiments verified that the conditions were within the exponential phase. The primers of cell cycle-regulated genes are listed in Table 1. The PCR product was analyzed by agarose gel electrophoresis and quantified using an image analyzer (Bio-Rad, Hercules, CA, USA). The result was verified in three independent experiments.

**Table 1:** Primers for RT-PCR analysis

Gene	Forward primer	Reverse primer
*cyclin B*	GCACTTTCCTCCTCCTCAA	CTTCGATGTGGCCATCTTG
*cyclin D1*	CTGTGCTGCGAAGTGGAAACCAT	TTCATGGCCAGCGGGAAGACCTC
*CDK1*	GATTCTATCCCTCCTGGTC	TAGGCTTCCTGGTTTCC
*CDK4*	CTGAGAATGGCTACCTCTCGATATG	AGAGTGTAACAACCACGGGTGTAAG
*CDK6*	CCGAGTAGTGCATCGCGATCTAA	CTTTGCCTAGTTCATCGATATC
*CDC25C*	GAACAGGCCAAGGCTGAAGC	GCCCCTGGTTAGAATCTTCC
*p53*	GAGGCGCTGCCCCCACCATGA	AGCTCTCGGAACATCTCGAAGC
*p16*	AGCCTTCGGCTGACTGGCTGG	CTGCCCATCATCATGACCTGG
*p21*	TTAGGGCTTCCTCCTGGAGGAGAT	ATGTCAGAACCGGCTGGGGATGTC
*p27*	CCTCTTCGGCCCGGTGGAC	TTTGGGGAACCGTCTGAAAC
*GAPDH*	GGGAAGGTGAAGGTCGGAGTC	AGCAGAGGGGGCAGA

### Western blot analysis

The protein expression of cell cycle-regulated genes was examined by Western blot. Cell extract was prepared using a non-denaturing lysis buffer. Protein concentration was determined using a Bio-Rad detergent-compatible protein assay kit (Bio-Rad). Samples (50-70 μg protein) were denatured in 5 × SDS-PAGE loading buffer and separated in 10% SDS-PAGE gels. The proteins were electro-transferred to nitrocellulose membranes followed by blocking with 5% (w/v) non-fat dry milk in Tris-buffered saline for 2 hours at room temperature. Membrane was probed with primary antibody at 1:400 dilution for 2 hours at room temperature and then washed three times with 0.1% Tween 20/PBS prior to incubation with an appropriate secondary antibody conjugated with peroxidase (Santa Cruz Biotechnology, Santa Cruz, CA, USA) for 1.5 hour. Signal detection was conducted using the enhanced chemiluminescence detection system (Bio-Rad). The blots shown are representative of three independent experiments. The primary antibodies to cyclin B, cyclin D1, CDK1, CDK4, CDK6, CDC25C, p53, p16, p21, p27, Rb, E2F, and GAPDH were purchased from Santa Cruz Biotechnology. To determine the levels of phosphorylated CDKs (pCDKs) and retinoblastoma (pRb) protein, the phospho-specific antibodies (Santa Cruz Biotechnology) targeting pCDK1 (Tyr15), pCDK4 (Tyr15), and pRb (Ser780) were used.

## Results

### Lobaplatin inhibited proliferation of human HCC cells

**Figure 1:**
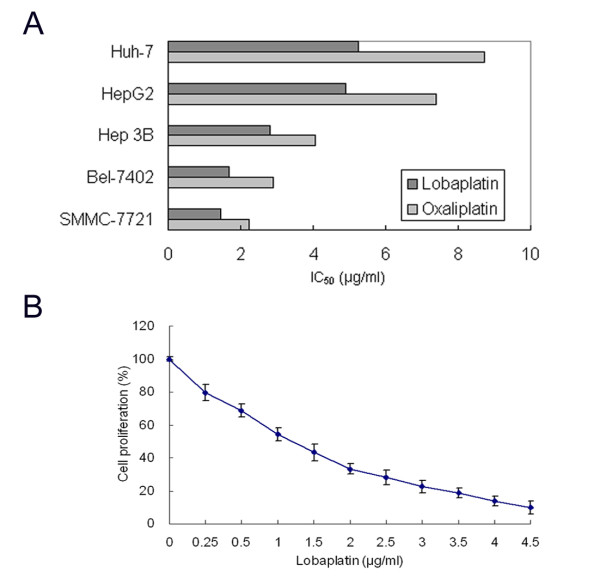
**Lobaplatin inhibited proliferation of human HCC cells**. **(A) **A comparison of lobaplatin and oxaliplatin in five human HCC cell lines. The IC_50 _value was determined using cell proliferation assay. **(B) **The dose-response curve of lobaplatin in SMMC-7721 cells. The cell proliferation rate of untreated cells was defined as 100% and that of treated cells was expressed as a percentage of the untreated cells. The data represented the mean ± standard deviations of three independent experiments.

As shown in Figure 1A, lobaplatin inhibited cell proliferation of cultured human HCC cell lines with the IC_50 _values (48 h) ranging from 1.45 to 5.22 μg/ml. The rank order of sensitivity was p53 wild-type SMMC-7721 > Bel-7402 > p53 null Hep 3B > p53 mutant Huh-7. The p53 wild-type HepG2 cell line showed a similar sensitivity to lobaplatin as the Huh-7 cells. In addition, lobaplatin appeared to have similar cytotoxicity profiles to oxaliplatin in these human HCC cell lines.

The dose-response curve of lobaplatin in SMMC-7721 cells was specially shown in Figure 1B. In a range of 0.25 to 4.5 μg/ml, lobaplatin inhibited cell proliferation of SMMC-7721 cells in a dose-dependent manner. The IC_50 _value of 1.45 μg/ml was chosen as a working concentration for subsequent cell cycle experiments in SMMC-7721 cells.

### Lobaplatin arrested cell cycle progression in G_1 _and G_2_/M phases time-dependently

**Figure 2:**
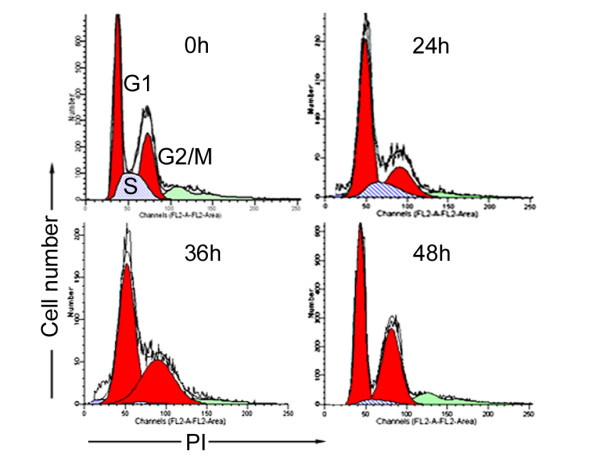
**Lobaplatin arrested cell cycle progression in G_1 _and G_2_/M phases time-dependently**. SMMC-7721 cells were treated with 1.45 μg/ml lobaplatin. In the course of treatment, cell cycle distribution was analyzed by FCM at 0, 24, 36, and 48 h. The profiles showed dual-variable plots of cell number versus PI uptake. G_1_, S, and G_2_/M cell populations were quantified.

The effect of lobaplatin on cell cycle distribution of SMMC-7721 cells was shown in Figure 2. After adjustment with their corresponding controls, the proportions of G_1_, S, and G_2_/M phases in cells treated with lobaplatin were 45.31, 22.88, and 31.81% at 0 h, 59.91, 11.92, and 28.17% at 24 h, 56.89, 2.83, and 40.28% at 36 h, and 53.80, 2.07, and 44.13% at 48 h, respectively. Under the induction of lobaplatin, accumulation of cells in G_1_ phase occurred from 24 to 48 h and G_2_/M phase arrest appeared from 36 to 48 h. A concurrent reduction of the cell population in S phase was observed. These data suggested that lobaplatin could arrest cell cycle progression in G_1 _and G_2_/M phases time-dependently.

### Lobaplatin down-regulated cyclin B, CDK1, CDC25C, pCDK1, and pCDK4

**Figure 3:**
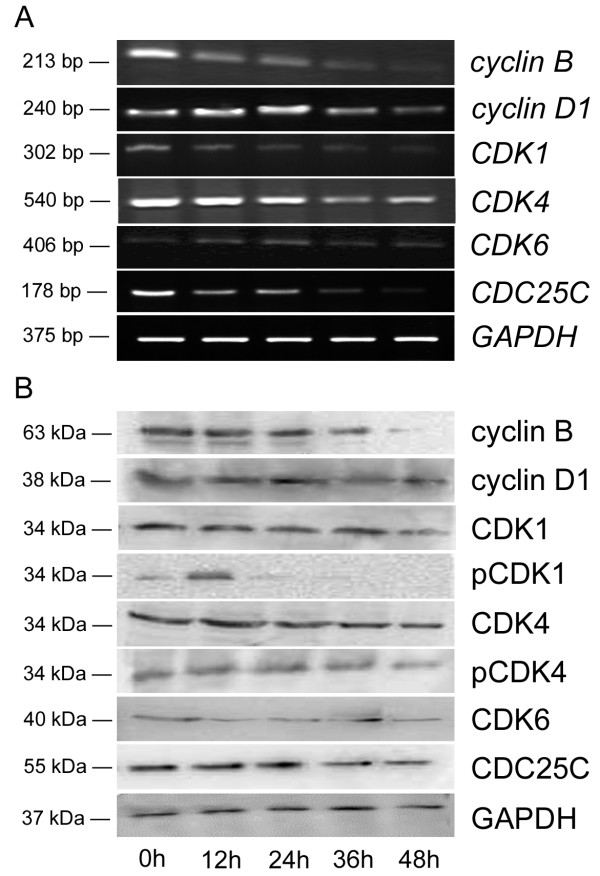
**Lobaplatin down-regulated cyclin B, CDK1, CDC25C, pCDK1, and pCDK4**. Expression of cell cycle-regulated genes was determined at 0, 12, 24, 36, and 48 h in SMMC-7721 cells after 1.45 μg/ml lobaplatin treatment. GAPDH as a control. **(A) **The mRNA level. **(B) **The protein level.

As shown in Figure 3A and Table 2, the mRNA levels of *cyclin B*, *CDK1*, and *CDC25C *phosphatase were moderately repressed at 24 h after lobaplatin treatment and significantly down-regulated at 36 and 48 h (changes > 2-fold). Meanwhile, the mRNA levels of *cyclin D1, CDK4, and CDK6 *were slightly enhanced or inhibited but the changes less than 2-fold compared to their controls. Lobaplatin did not appear to affect the mRNA levels of *cyclin D1, CDK4, and CDK6*.

**Table 2:** Genes/GAPDH ratio at the mRNA level (The densitometric data are presented as fold changes as compared with their corresponding controls)

Treatment time	*cyclin B*	*cyclin D1*	*CDK1*	*CDK4*	*CDK6*	*CDC25C*	*P53*	*p16*	*p21*	*p27*
0 h	1	1	1	1	1	1	1	1	1	1
12 h	0.89	1.47	0.83	1.21	1.10	0.51	3.03	1.22	2.05	1.26
24 h	0.53	1.36	0.53	0.95	1.14	0.54	2.69	1.85	2.50	1.32
36 h	0.18	0.87	0.25	0.52	1.18	0.21	1.30	1.59	3.14	2.05
48 h	0.09	0.59	0.13	0.53	1.04	0.12	0.79	1.77	3.64	1.95

The protein expression of genes mentioned above was generally consistent with the mRNA expression. As shown in Figure 3B and Table 3, the fold changes of genes at the mRNA level were further confirmed by the protein level. Moreover, lobaplatin could regulate the phosphorylation status of CDKs and significantly reduce both pCDK1 after 24 h of treatment and pCDK4 after 36 h.

**Table 3:** Genes/GAPDH ratio at the protein level (The densitometric data are presented as fold changes as compared with their corresponding controls)

Treatment time	cyclin B	cyclin D1	CDK1	pCDK1	CDK4	pCDK4	CDK6	CDC25C	p53	p16	p21	p27	E2F	Rb	pRb
0 h	1	1	1	1	1	1	1	1	1	1	1	1	1	1	1
12 h	0.94	1.17	0.79	1.50	1.15	0.75	1.21	1.25	4.16	0.87	2.15	1.58	1.14	0.98	1.01
24 h	0.47	1.28	0.59	0.39	0.95	0.51	1.04	1.58	2.41	1.66	5.32	2.04	0.81	0.39	0.55
36 h	0.26	0.77	0.23	0.15	0.56	0.36	1.11	0.63	1.57	1.39	7.00	3.00	0.46	0.45	0.29
48 h	0.07	0.64	0.09	0.02	0.65	0.19	1.19	0.47	0.63	1.74	5.63	1.96	0.40	0.39	0.34

### Lobaplatin up-regulated p53, p21, and p27

**Figure 4:**
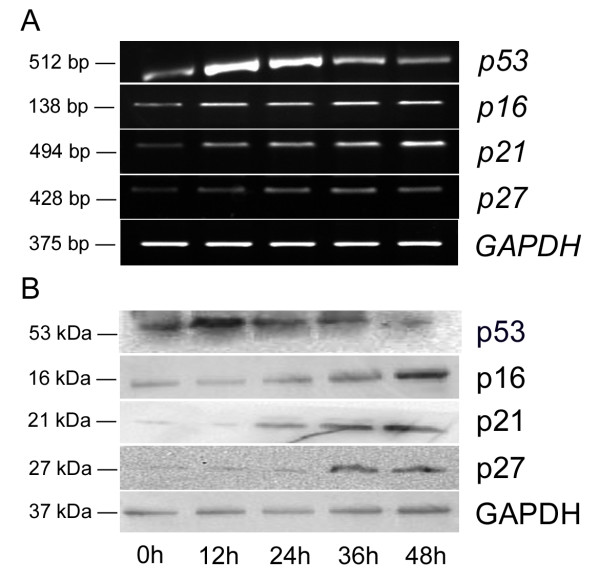
**Lobaplatin up-regulated p53, p21, and p27**. Expression of p53 and CDK inhibitors was determined at 0, 12, 24, 36, and 48 h in SMMC-7721 cells after 1.45 μg/ml lobaplatin treatment. GAPDH as a control. **(A) **The mRNA level. **(B) **The protein level.

The effect of lobaplatin on p53 and CDK inhibitors (p16, p21, and p27) was subsequently examined at both the mRNA and protein levels (Figure 4, Table 2, 3). The results indicated that the expression of p53 was significantly increased within 24 h after lobaplatin treatment and p27 was up-regulated at somewhat later time points. The expression of p21 continued to be up-regulated and reached a peak of 7-fold increase at 36 h at the protein level. No significant change of p16 was found after lobaplatin treatment.

### Lobaplatin down-regulated Rb, E2F, and pRb

**Figure 5:**
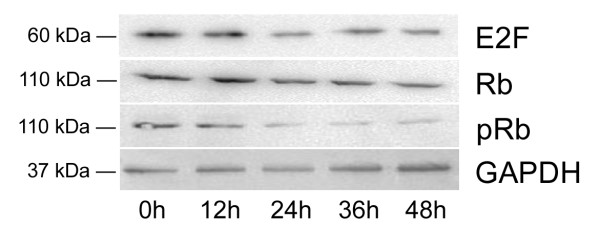
**Lobaplatin down-regulated Rb, E2F, and pRb**. The protein expression of Rb, E2F and pRb was examined at 0, 12, 24, 36, and 48 h in SMMC-7721 cells after 1.45 μg/ml lobaplatin treatment. GAPDH as a control.

During the lobaplatin treatment, the significant down-regulation of Rb appeared at 24 h followed by a persistent low level while its phosphorylation status (pRb) was significantly reduced in the late course of treatment. E2F also became significantly down-regulated after 36 h of lobaplatin treatment (Figure 5 and Table 3).

## Discussion

The present study aimed at evaluating cytotoxicity of lobaplatin in human HCC cells *in vitro*. Among the five human HCC cell lines used, SMMC-7721 was the most sensitive one to lobaplatin and hence was selected as the cell model to reveal the underlying cytotoxic mechanisms of lobaplatin in terms of cell cycle kinetics. The results suggested that (i) lobaplatin could inhibit the proliferation of human HCC cells through arresting cell cycle progression in G_1 _and G_2_/M phases; (ii) The cell cycle arrest on human HCC cells induced by lobaplatin might be associated with the down-regulation of CDK1/cyclin B and Rb/E2F complexes and the up-regulation of CDK inhibitors.

Lobaplatin has shown favorable activity in various types of cancers including breast, oesophageal, lung, and ovarian cancers as well as CML [8]. In this study, lobaplatin exhibited evident cytotoxicity to human HCC cells. Interestingly, the p53 wild-type SMMC-7721 and Bel-7402 were the most sensitive cell lines to lobaplatin than Huh-7 which was p53 mutant. It indicates an important role for p53 phenotype in response to lobaplatin. However, the fact that p53 wild-type HepG2 cell line was resistant to lobaplatin suggests p53 phenotype is not the sole determinants of sensitivity to lobaplatin for human HCC cells. Moreover, characterized by p53 phenotype in these HCC cell lines, lobaplatin appeared to have similar cytotoxicity profiles to oxaliplatin which was active for advanced HCC patients [5,6]. The results indicate that lobaplatin may have potential value for the management of human HCC.

From the viewpoint of cell cycle, cytotoxicity of lobaplatin might be due to its ability to arrest cell cycle progression in our study. Upon incubation with lobaplatin, SMMC-7721 cells were continuously arrested in G_1_ phase after 24 h of treatment. It is well known that the complexes of CDK4, 6/cyclin D play an important role in G_1_-S transition by phosphorylating Rb [12,13]. As a consequence of Rb phosphorylation, E2F is released from the Rb/E2F complex, thereby activating the expression of the genes that are required for S phase transition [14]. Our results showed that the expression of CDK4, 6/cyclin D1 complexes was not affected by lobaplatin. Thus, there may be other mechanisms contributed to G_1 _phase arrest in this study. For the reason that the activity of CDKs is negatively controlled by binding CDK inhibitors to CDK/cyclin complexes [15], we examined the expression of CDK inhibitors both at the mRNA and protein levels. The results indicated that lobaplatin drastically enhanced the expression of p21 and p27, suggesting that CDKs activity may be inhibited by these two CDK inhibitors. Furthermore, lobaplatin down-regulated the expression of Rb/E2F complex and consequently inhibited the expression of E2F target genes. Meanwhile, the changes of pCDK4 and pRb were revealed in accordance with this cell cycle variation.

The cell cycle analysis in this study revealed a prominent G_2_/M phase arrest in the late course of lobaplatin treatment. G_2_-M transition is partly governed by the activity of CDK1, which is positively regulated by cyclin B [16]. CDK1 activation is also controlled by dephosphorylation at Tyr15 by CDC25C phosphatase [16,17]. Lobaplatin significantly down-regulated cyclin B, CDK1, and CDC25C as well as pCDK1. Absence of cyclin B and CDK1 after 36 h of treatment might have contributed to G_2_/M phase arrest as a late event. The reduced expression of CDC25C may have contributed to the lower CDK1 activity.

As an essential cell cycle regulator, the p53 tumor suppressor plays an important role in the cellular response to platinum agents. For example, 1,2-diaminocyclohexane-acetato-Pt could arrest the wild-type p53 cells in G_1 _phase and the mutant p53 cells in G_2_/M phase in ovarian cancer [18]. P53 transcriptionally activates a series of genes involved in both G_1_-S and G_2_-M transitions in response to genotoxic stress [19,20]. Among these genes, p21 is a well-established negative regulator of G_1_-S transition [19]. It also inhibits the CDK1/cyclin B complex and keeps G_2 _arrest maintenance [20]. In the present study, lobaplatin induced a rapid accumulation of p53 which occurred within 24 h of lobaplatin treatment. Consistent with this finding, p21 was strongly up-regulated with a 7-fold increase at 36 h after lobaplatin treatment. The data suggest that the p53-p21 pathway may contribute to G_1 _and G_2_/M cell cycle arrests in this p53 wild-type SMMC-7721 cells [21,22].

Being similar to cisplatin, lobaplatin induces intra-strand DNA-Pt crosslinks [23] but somewhat less efficiently [24]. Lobaplatin shows incomplete cross-resistance with cisplatin [23] which suggest the former might have an underlying action mechanism different from the latter. Cisplatin can reduce the DNA synthesis rate with a subsequent accumulation in S phase followed by G_2_/M phase arrest [25-27]. The results in our study lobaplatin arrested SMMC-7721 cells in G_1 _and G_2_/M phases demonstrate the existence of a different action mechanism of lobaplatin. Oxaliplatin, another third-generation platinum compound, could activate G_1_-S checkpoint and block G_2_-M transition completely in p53 wild-type HCT-116 colon carcinoma cells [28]. As revealed in this study, the effect of lobaplatin on cell cycle seems similar to that of oxaliplatin. Further studies should be conducted to examine whether the effect of lobaplatin on G_1_-S transition is associated with its incomplete cross-resistance with cisplatin.

In conclusion, the present study demonstrated the encouraging efficacy of lobaplatin against human HCC *in vitro*. Lobaplatin could arrest cell cycle in G_1 _and G_2_/M phases which was possibly associated with the down-regulation of cyclin B, CDK1, CDC25C, pCDK1, pCDK4, Rb, E2F, and pRb, and up-regulation of p53, p21, and p27. These alterations of cell cycle kinetics might contribute to a better understanding for cytotoxicity of lobaplatin and facilitate its potential use for the management of HCC.

## Competing interests

The authors declare that they have no competing interests.

## Authors' contributions

QW and SKQ were responsible for research design and manuscript preparing. FMT, CJC, and RW performed the experiments and analyzed the data. All authors have read and approved the final manuscript.
